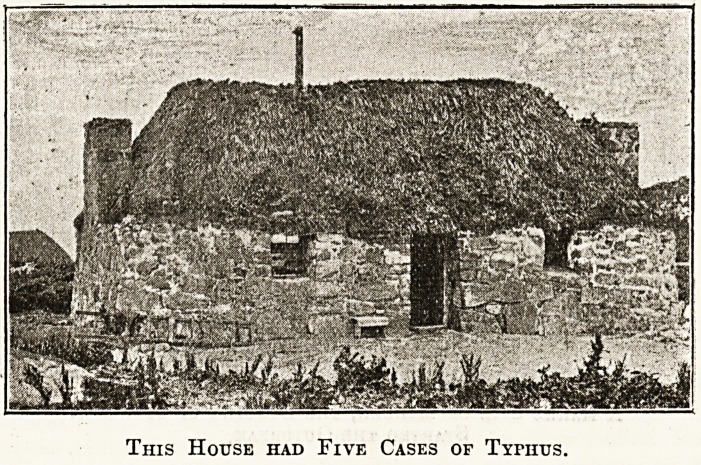# A Recent Outbreak of Typhus Fever

**Published:** 1913-02-08

**Authors:** Wm. C. Burns

**Affiliations:** late Medical Officer of Health, South Uist.


### A RECENT OUTBREAK OF TYPHUS FEVER.

By WM. C. BURNS, M.B., C.M.Edin., late Medical Officer of Health, South Uist.

As typhus fever is not nowadays a disease that many doctors or nurses have an opportunity of seeing, perhaps a brief account of an outbreak with which I recently had to deal may be of some interest to your readers. The outbreak occurred in Eriskey, a small island of the Outer Hebrides, known to fame as the landing-place of Prince Charlie when he came to head the famous rising of the "Forty-Five."

A most interesting point in connection with the outbreak was that, owing to the complete isolation of the island and the fact that it was practically impossible for infection to come from any outside sources, we were able to get at the *fons et origo* of the disease.

Thirteen years previous to this outbreak there had been an epidemic of typhus in the island. The inmates of one house had all perished, and the succeeding tenant of the croft, fearing to occupy a house in which this had occurred, built a new abode some little distance away, leaving the old house and its surroundings in disuse. The kitchen midden of this old house, a shallow mound chiefly consisting of peat ashes, had lain undisturbed during those thirteen years. On this occasion, however, being short of manure, the woman of the house dug it up and spread it on her potato patch. Within fourteen days she was down with typhus fever. All the other cases were proved "contacts" from this. The kitchen midden was situated on a small mound within a few yards of the seashore, drenched with salt spray during the greater part of the year, and at all times fully exposed to the action of sun, wind, and rain.

According to Nicolle and other workers body-lice are said to be the natural carriers of the infection of typhus, but under conditions such as I have described I think we may safely put aside lice as being the exciting cause of this outbreak. Lice may be the most common "spreaders" of the disease, but I think this case clearly proves that they in no sense stand in the same relation to it that mosquitoes do to malaria, and that the virus of typhus is capable of surviving for quite indefinite periods, in positions and under conditions, that would seem entirely to preclude the agency of lice.

Believing that the *Pediculus corporis* would be an active spreader of the disease, and knowing it to be very much in evidence in some of the houses, I took the precaution of using a very liberal supply of insecticide. The formalin spray was also freely used about the walls, floors, and beds, and whether or not it acted as a prophylactic to the attendants, it at least gave a feeling of freshness and coolness to the close and stuffy rooms, and seemed to be greatly appreciated by the patients themselves.

It has always been laid down as a maxim in the management of typhus that abundance of fresh air should be allowed to circulate through the room, but when one has three patients in a room barely nine feet square, as we had in one house, with a window consisting of a single fixed pane of glass, the ventilation problem becomes rather a difficult one, and we found in practice that frequent spraying had a very beneficial effect.

X MARKS SITE OF MIDDEN, THE DIGGING UP OF WHICH STARTED THE OUTBREAK.

As long as a patient was sufficiently sensible to use them we found that sucking formalin lozenges was most useful for keeping the mouth clean and comfortable. When he became stuporous or unconscious, of course the nurse had to swab out the mouth in the usual way.

Insomnia was a very marked feature in nearly all cases, and had to be most persistently combated. We found that the action of whatever hypnotic was being administered seemed to be considerably aided by a tepid sponging of the patient with a mild antiseptic wash. In the unconscious cases also sponging seemed to give considerable temporary relief and greatly modified the incessant, weary tossing.

Getting the patient to take sufficient nourishment of any kind was a great difficulty, and it was a point that had to be most conscientiously insisted upon in order to keep up the strength as well as possible against the period of utter depression and weakness which supervened in all cases.

I cannot argue on the relative merits of treatment with or without alcohol, because in all cases when the pulse began to falter and the strength seemed failing fast I gave alcohol and milk and, as far as I could judge, with distinct benefit to the patient. It may, of course, be said that I am not in a position to prove that they would not have done equally well, or perhaps better, without it. Anyhow, it was according to ancient practice and I did not feel justified in withholding it. As regards diagnosis, there was no difficulty when once suspicion had been aroused, but I must confess that on my first visit to the first case I thought it one of simple influenza. The history of a chilly onset with subsequent fever, headache, body pains, and general malaise seemed, in the absence of any cause for suspicion, to point to that complaint. It was only on a subsequent visit that the appearance of a petechial rash, together with the increased gravity of her symptoms, led me to a knowledge of the true nature of the disease. In another case I should certainly have put it down to heat-stroke but for the prevalence of typhus at the time. This elderly woman had been working in the field all day exposed to a very hot sun. She complained of sudden vertigo and faintness and, after getting into her house, was seized with violent vomiting and prostration. When I saw her some eight hours afterwards she was in a state of high fever and partial delirium, and had all the appearance, when taken with the history of the onset, of a person suffering from a severe sunstroke. This proved to be an excessively severe case; she seemed to be absolutely overwhelmed with the severity of the toxins and died in a raging fever and delirium on the fourth day. In contrast to this case there was one woman who had all the typical symptoms, yet in so mild a degree that she refused to go to bed, and passed the whole day sitting in the sun at her cabin door. A very typical symptom that all complained of, especially in the earlier stages, was a curious pain, neither altogether abdominal nor yet thoracic. It seemed to me as if it followed the attachment of the diaphragm. Some compared it to a tight constricting band, some to a strong expansive force as if something were forcing out the edges of the ribs, but at all events one and all experienced it. The petechial spots were not well marked in all cases, and in some only the typical sub-cuticular mottling in the flanks could be noticed. Contrary to the usually received belief, we did not find that the worst cases showed the most abundant rash. One woman, whose legs, thighs, and back were simply covered with dark purpuric spots, had only a rather moderate attack, although rather lingering, both as to time of crisis and subsequent convalescence. An early and well marked symptom was a very noticeable slowness of mind long before any confusion of ideas occurred.

In nearly all cases there was the well marked dusky hue of countenance and congestion of the conjunctivæ so typical of the disease. But in the case of the woman with the profuse purpuric mottling the eyes and the countenance remained quite clear for the first ten days. They all had rather a troublesome cough, and bronchitic rales were well heard over chest and back. Some of them had a considerable amount of hypostatic congestion. Luckily we had no examples of delirium ferox, as the nurse would have had small assistance in dealing with them. The delirium was in all cases of a low muttering type. The " picking of the bedclothes sign " justified its fatal reputation. In the case of one man who was busy at this occupation when I went in to see him, the nurse's report, his chart, and his appearance seemed to imply that he had favourably passed the crisis, and he lived for several days afterwards, yet down he went in the end.

This House had Five Cases of Typhus.

The first case was one of the woman with the well marked rash, and showed a remarkably long continued sub-normal temperature after the crisis was passed.

The second case was a very typical average case with recovery, and the third appeared to pass the crisis successfully, but died of exhaustion the fifth day afterwards.

Owing to the prompt measures taken by the sanitary authorities, backed by the automatic isolation of all " suspects " by the terror-stricken inhabitants when the nature of the disease became known, the outbreak was strictly limited to direct contacts of first and second cases. There were in all ten cases with three deaths. The fatal cases were people of forty-nine years and over; the survivors thirty-one and under. They were all healthy people accustomed to an active outdoor life, and the conditions of treatment, feeding, nursing, etc., were the same in all.

The extreme dislike of relatives and friends to go near any of the sufferers, while it certainly tended to limit the extent of the outbreak, was not without its disadvantages, as the nurse could get no assistance whatever from the friends, and had it not been for the ungrudging help of the Catholic priest of the island would have been very hard put to it indeed. And when a death occurred the priest, the doctor, and the nurse had to become amateur undertakers, prepare and coffin the body, screw down the lid, and carry the shell out to the grass, when, after it had been liberally sprinkled with disinfectants, the friends gingerly approached and carried it off for burial.

The ordinary methods of disinfection as applied to these old houses are altogether ineffectual. The sanitary authorities seem to be powerless in the matter. They can order all the furnishings and personal effects of the inmates to be burned, and will grant compensation for everything destroyed by their order; but have no power to interfere with the house itself.

A typical old "black house" as it is called, like the one in which five cases occurred, is dry-stone built—*i.e.* without lime, the spaces between the stones being stuffed with peat or mud. The roof is thatched with straw or heather laid on the bare rafters, and the floor is simply trodden earth. The re-thatching of such a house was known to be the starting-point of a serious epidemic which occurred in the Island of Skye some years ago, full details of which were published by the late Dr. Dewar, of Portree. Nothing but burning to the ground would be of any use, and yet these old houses saturated with the virus of typhus are allowed to stand as a perpetual menace to future generations of occupiers. When the present survivors give place to successors who have not been rendered immune, some accidental stirring up of the poison is almost certain to lead to another outbreak, when this lovely little island will again become the scene of quite avoidable suffering and death.

## Figures and Tables

**Figure f1:**
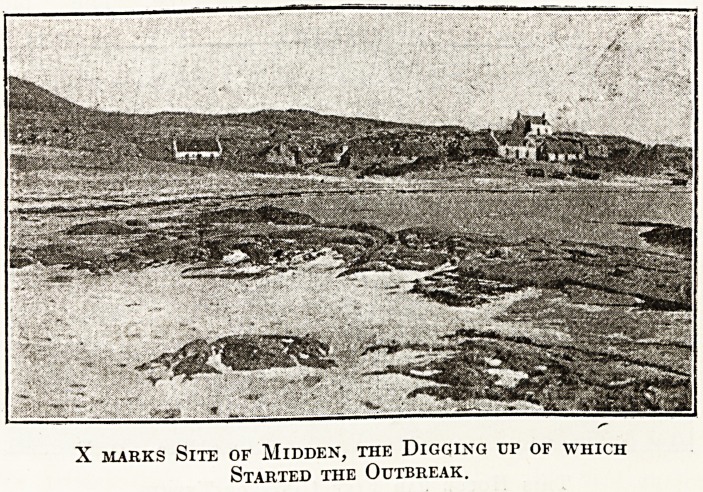


**Figure f2:**